# Marital transitions and associated changes in fruit and vegetable intake: Findings from the population-based prospective EPIC-Norfolk cohort, UK

**DOI:** 10.1016/j.socscimed.2016.04.004

**Published:** 2016-05

**Authors:** Johan L. Vinther, Annalijn I. Conklin, Nicholas J. Wareham, Pablo Monsivais

**Affiliations:** a London School of Hygiene & Tropical Medicine, University of London, UK; b Danish Cancer Society, Documentation & Quality, Copenhagen, Denmark; c Centre for Diet and Activity Research (CEDAR), Medical Research Council Epidemiology Unit, University of Cambridge, UK; d WORLD Policy Analysis Center, UCLA Fielding School of Public Health, Los Angeles, CA, USA

**Keywords:** Marital status, Marital termination, Fruit and vegetable, Gender, Social ties

## Abstract

**Background:**

Diet is critical to health and social relationships are an important determinant of diet. We report the association between transitions in marital status and healthy eating behaviours in a UK population.

**Methods:**

Longitudinal study of middle-age and older adults 39−78y (n = 11 577) in EPIC-Norfolk, a population-based cohort, who completed food frequency questionnaires in 1993–97 and 1998–2002. Multivariable linear regression analyses assessed gender-specific associations between five categories of marital transitions and changes in quantity (g/d), and variety (no/month) of fruits or vegetables.

**Results:**

In 3.6 years of follow-up and relative to men who stayed married, widowed men showed significant declines (mean difference, 95% CI) in all four indicators of healthy eating including fruit quantity (−47.7, −80.6 to −14.9 g/d), fruit variety (−0.6, −1.1 to −0.2 no/month), vegetable quantity (−27.7, −50.5 to −4.9 g/d), and vegetable variety (−1.6, −2.2 to −0.9 no/month). Men who were separated or divorced or who remained single also showed significant declines in three of the indicators. Among women, only those who became separated/divorced or stayed single showed declines in one indicator, vegetable variety.

**Conclusion:**

Unhealthy changes to diet accompanying divorce, separation and becoming widowed may be more common among men than women. Moreover, deterioration in fruit and vegetable intakes was more apparent for variety rather than quantity consumed. Programmes to promote healthy eating among older adults need to recognise these social determinants of diet and consider prioritising people who live alone and in particular men who have recently left relationships or who have been widowed.

## Highlights

• Both quantity and variety of fruit and vegetable (F&V) intake are associated with lower risk of chronic disease.
• F&V intake is associated with marital status, but less is known about marital transitions and diet, particularly by gender.
• We studied marital transitions and changes in quantity and variety of F&V in middle-age and older UK men and women.
• For men more than women, becoming widowed or separated was linked to declines in F&V quantity and variety.
• Interventions to promote healthy eating may be needed for men experiencing separation, divorce or death of a spouse.

## 1 Introduction

Non-communicable diseases present a significant societal challenge to both high-income and low-income countries, with a growing health and economic burden of cardiovascular diseases, type 2 diabetes, and other chronic conditions (World Health Organization, 2013). Diet is a key modifiable risk factor for multiple chronic diseases and thus many national and international policies acknowledge the importance of supporting individuals in achieving a healthy balanced diet (World Health Organization, 2003). Among recognized determinants of diet are a person's social relationships (Conklin et al., 2014a, Holt-Lunstad et al., 2010).

Evidence from observational studies in many countries suggests that different types of social relationships are important influences on food consumption. Specifically, individuals who are married report higher fruit and vegetable (FV) intake than persons who are not married, and those who have lost a partner appear to have poorer nutrition than those without experiences of bereavement (Roos et al., 1998, Rosenbloom and Whittington, 1993). Moreover, the strength of association between marital status and healthy eating behaviours was shown to be stronger for men than women, suggesting that the benefits of marriage or partnered relationships may differ by gender (Conklin et al., 2014a). Much of the work on marital status and diet has come from cross-sectional studies and less is known about how changes in marital status relate to changes in diet over time.

Only two prospective studies in the US have investigated associations between marital transitions and dietary behaviours (Eng et al., 2005, Lee et al., 2005). Results showed that marital termination for men was linked to adverse changes in dietary behaviours, while for women the association with diet was weaker. Such findings suggest that the benefits of marriage and the effects of marital termination affect dietary behaviour in men and women differently. Those US studies examined each gender in separate occupational cohorts using different methodologies. Thus there is a need to examine longitudinal evidence from a population-based sample comprising both women and men in the UK.

The aim of this study was to examine the prospective relationship between marital transitions and healthy dietary behaviours in adult women and men in a population-based cohort. Specifically, we examined quantity and variety of fruit and vegetable intake. Intake of low quantities of fruit and vegetable is associated with higher risk mortality due to cardiovascular disease (Crowe et al., 2011) and cancer (Oyebode et al., 2014), while independent of quantity, low variety is associated with higher risk of diabetes (Cooper et al., 2012) and some cancers (Jeurnink et al., 2012). We hypothesised that marital transitions would be associated with changes in dietary behaviours differently for women and men.

## 2 Methods

### 2.1 EPIC-Norfolk cohort study

We used data from the prospective EPIC-Norfolk study. The EPIC-Norfolk study aimed to quantify the contribution of nutrition and other determinants of chronic diseases in middle and later life. It recruited from age-sex registers of general practices in Norfolk, UK, which is a geographical circumscribed area with little outward migration and a population mainly served by one District General Hospital, as described in detail elsewhere (Day et al., 1999). The Norwich local research ethics committee approved the study, and all volunteers gave written informed consent.

### 2.2 Study population and analytic sample

At cohort entry, a total of 25 639 participants aged 39–79 attended the first health check (HC1) of whom 54.7% were women and the overall mean age was 59.2 years. Participants also completed a Food Frequency Questionnaire (FFQ) and detailed Health and Lifestyle Questionnaire in which they reported their occupation, educational level, health status and chronic health conditions (Day et al., 1999). A second health check (HC2), an average of 3.6 years following entry, was attended by 15 786 participants, among whom 12 331 completed a second FFQ. This study's analytic sample (n = 11 577) therefore comprised cohort participants providing marital status and food consumption data at baseline and follow-up, as well as key covariates (see Supplementary Figure S1). The restriction of the cohort for the purpose of our analyses resulted in a sample that, relative to the total cohort, was slightly more highly-educated and from higher social classes and less-likely to smoke and in better overall health (Supplementary Table S1). However, diet appeared to be similar between the analytic sample and full sample.

### 2.3 Marital status and marital transitions

Marital status was obtained from the Health and Lifestyle Questionnaire with five response categories (single, married/living as married, widowed, separated and divorced) at HC1 and HC2. We used data from both time-points to construct a new variable to assess marital transitions, which we defined according to a five-category classification used in previous studies (Eng et al., 2005, Lee et al., 2005). Marital transition categories included: *remain married* (married at both time-points, reference group), *remain unmarried* (single, divorced or widowed at both time-points), *became separated/divorced* (married at baseline and separated or divorced at follow-up), *became widowed* (married at baseline and widowed at follow-up) and *became married* (single, widowed, divorced or separated at baseline and married at follow-up).

### 2.4 Dietary assessment and healthy dietary behaviour outcomes

Habitual diet was assessed with a semi-quantitative FFQ—previously validated by comparing to a 16-day weighed food record (Bingham et al., 1994), and to nutrient biomarkers (Bingham et al., 2000). Individuals reported their intake of 11 fruits (whole item or medium serving) and 26 vegetables (medium serving) over the previous year, with nine standard response categories between “never or less than once/month” and “six times per day or more”. The average daily consumption of each fruit and vegetable item (g/d) was estimated from self-reported frequencies and imputed standard portion sizes using the FFQ EPIC Tool for Analysis software (Mulligan et al., 2014) developed from a previous programme used to estimate average daily intakes of energy, nutrients and food groups (Welch et al., 2005). We excluded FFQ respondents with implausible estimated energy intakes (n = 124), defined as top and bottom 0.5 percentile of energy intake relative to basal metabolic rate values (Welch et al., 2005).

Dietary intakes and changes in dietary behaviours were characterised in terms of energy intake, percent energy from protein, carbohydrates and fat, and four characteristics of fruit and vegetable intake, as indicators of healthy eating habits (see below) and alcohol intake in units/week. Fruit and vegetable intakes were characterised by: (1) fruit quantity (g/d), (2) fruit variety (no/m), (3) vegetable quantity (g/d) and (4) vegetable variety (no/m). We summed the total amount of fruits or vegetables that were reported to have been consumed, constructing two separate quantity variables (g/d) at baseline and follow-up. Variety of fruit or vegetable intake was a sum of the total number of unique items consumed, irrespective of quantity (>0 g/d), which corresponded to frequency responses of at least 1–3/month. Continuous scores for variety were derived at baseline and follow-up, and followed similar approaches previously demonstrated in this cohort for reduced risk of chronic diseases (Cooper et al., 2012, Jeurnink et al., 2012). Variety scores reflected the two-week timeframe for a person to exhaust the range of different items in their food repertoire (Drewnowski et al., 1997). The reproducibility and validity of the variety scores for nutritional adequacy in older populations is further demonstrated in other studies (Drewnowski et al., 1997, Bernstein et al., 2002). Change in quantity and in variety was calculated as the difference between follow-up and baseline.

### 2.5 Data analysis

Descriptive statistics were used to analyse socio-demographic characteristics and eating behaviours across the five categories of marital transition. Multivariable linear regression analyses were performed to examine the gender-specific effect of marital transitions on changes in healthy eating behaviours adjusting for known confounders. Covariates included were age, change in total energy intake, education, occupation-based social class, and baseline variable for a given outcome (e.g. baseline *fruit quantity* for analyses of *change in fruit quantity*), an approach used by others (Lee et al., 2005). For all four fruit and vegetable outcomes, regression models that contained interaction terms for gender by marital transition indicated a significant interaction, suggesting that associations between marital transition and dietary changes differed for men and women. Subsequently, gender-stratified analyses provided separate estimates of dietary changes from marital transitions for women and men separately. We present results as beta-coefficients and 95% confidence intervals (with p-values) relative to the group remaining married, which is essentially a difference-in-difference measure.

### 2.6 Sensitivity analyses

Sensitivity analyses tested, separately and combined, for potential confounding from other lifestyle factors at baseline (smoking status, alcohol intake, and physical activity), waist circumference, general health status, depression and quantity (for variety outcomes) or variety (for quantity outcomes). Sensitivity analysis also further adjusted for changes in body weight and for alternative specification of the *remained unmarried* category. Because marital transitions and changes in diet might both be influenced by chronic disease conditions, we conducted further analyses in which we excluded obese participants (BMI 30 kg/m2 and higher) or those who reported having common chronic diseases at baseline. These were chronic diseases were diabetes, myocardial infarction, stroke or cancer. All analyses were conducted in SPSS version 19.

## 3 Results

The analytical sample of the EPIC-Norfolk cohort study used here included 57% women and had a mean age of 59 years. Fifteen percent of the participants were educated to degree-level or higher, with a greater proportion of men than women attaining this level of education. At baseline, approximately 8% were current smokers and 84% reported being in good/excellent general health. The mean BMI for the sample at baseline was 26.0 kg/m^2^ and estimated fruit and vegetable intake was 257 and 275 g/day, respectively. Overall, 83% of the participants were married at baseline but more men (89%) than women (78%) were married. By contrast, a higher percentage of women (10%) than men (3%) were widowed. A similar proportion of women and men were single (4%) at baseline.

Table 1a, Table 1b shows the distribution of demographic characteristics and crude reported intake of fruit and vegetables across different marital transition categories for men and women, followed-up for 3.6 years. Gender differences were observed in the distribution of all socio-demographic variables, except BMI, across different categories of marital transition. For example, men who remained unmarried were more likely to be from lower social classes and lower educational attainment. For both men and women, relatively high percentages of participants with moderate to poor self-reported health at baseline were found in those who became widowed or remained unmarried. Among men, self-reported depression at baseline varied sharply across groups with the highest percentage of depression in men who went on to separate or divorce. For women, lower social class and educational attainment was found more among those who became widowed. On average, both women and men who became separated/divorced consumed the lowest quantity, and variety, of fruits at baseline. However, the lowest quantity of vegetable intake was observed in men who remained unmarried but in women who became separated/divorced. Both women and men who remained unmarried reported the lowest variety of intake of vegetables.

### 3.1 Fruit quantity and variety

Marital transitions were associated with significant changes in fruit intake for men. Table 2 shows estimated mean changes in fruit consumption in four marital transition categories relative to those who remained married for men and women separately. Men who became widowed had a mean (95% CI) change in fruit intake of −46.0 (−82.5, −9.6) g/day, and this estimate was not substantially altered by progressive adjustment for a number of potential confounders. The analysis for fruit variety (not shown) showed a similar pattern as fruit quantity. Below, only models with all covariates included are presented.

**Fig. 1:**
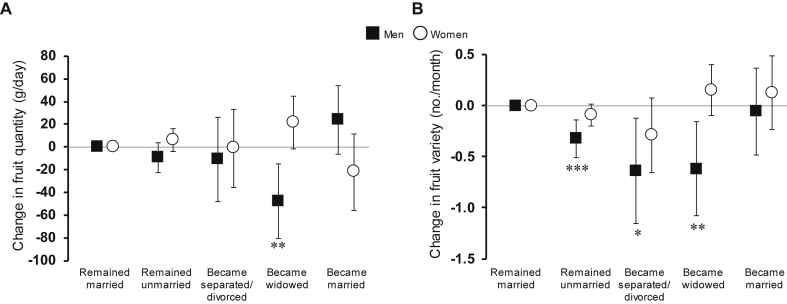
Relative change in quantity (left) and variety (right) of reported intake of fruits associated with 3.5-year marital transitions in the EPIC-Norfolk study by gender. Mean changes and 95% confidence intervals are unstandardised beta coefficients from regression models adjusted for age, change in total energy intake between baseline and follow-up, education, occupation-based social class, and baseline fruit quantity or variety for each analysis respectively. *P-values* for difference from remained married within each gender are indicated by *** <0.001; **<0.01; *<0.05.

Fig. 1 illustrates the change in fruit quantity and in fruit variety for women and men reporting different marital transitions, compared to those who remain married. Men who became widowed significantly reduced both their amount and variety of fruits consumed. Relative to men who stayed married, those who became widowed reduced their quantity (−47.7 [−80.6, −14.9] g/day) and variety (−0.6 [−1.1, −0.2] no/month) of fruit intake. Men who separated/divorced showed similar reductions in fruit variety (−0.6 [−1.2, −0.1] no/month) and men who remained unmarried showed reductions in variety of −0.3 (−0.5, −0.1) no/month. Among women, marital transitions were not associated with significant changes in either fruit quantity or variety.

### 3.2 Vegetable quantity and variety

**Fig. 2:**
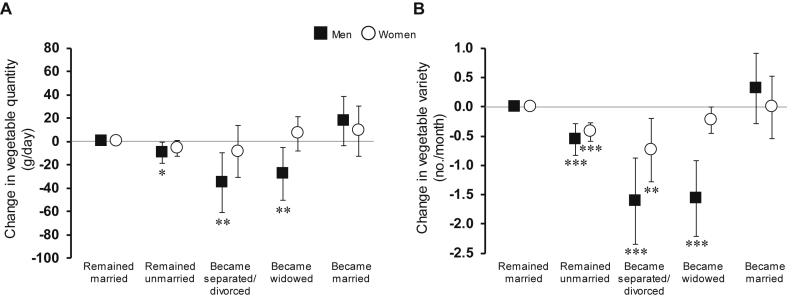
Relative change in quantity (left) and variety (right) of reported intake of vegetables associated with 3.6-year marital transitions in the EPIC-Norfolk study by gender. Mean changes and 95% confidence intervals are unstandardised beta coefficients from regression models adjusted for age, change in total energy intake between baseline and follow-up, education, occupation-based social class, and baseline vegetable quantity or variety for each analysis respectively. *P-values* for difference from remained married within each gender are indicated by *** <0.001; **<0.01; *<0.05.

Results indicated significantly reduced vegetable quantity in men who remained unmarried (−9.7 [−18.9, −0.5]), became widowed (−27.7 [−50.5, −4.9]), or separated/divorced (−35.0 [−60.8, −9.2]), compared to men remaining married (Fig. 2). No significant change in vegetable quantity was observed in women for any marital transition.

There was also significantly less vegetable variety reported in men who remained unmarried (−0.6 [−0.8, −0.3]), became separated/divorced (−1.6 [−2.3, −0.9]) or became widowed (−1.6 [−2.2, −0.9]), compared to men remaining married. For women, vegetable variety was also significantly reduced in those remaining unmarried (−0.4 [−0.6, −0.3]) and becoming separated/divorced (−0.7 [−1.3, −0.2]).

Further stratifying the transition group, *remaining unmarried*, did not reveal substantial differences among men and women who remained unmarried from having reported being single, divorced/separated or widowed at baseline (Supplementary Table S2). These analyses also indicated few differences among groups in terms of changes in energy or macronutrient intakes. Only men and women remaining widowed showed significant reductions in energy intake relative to their counterparts remaining married. Men in this group also showed significant increases in the percent energy from fat in their diets. Men showed no significant differences in change in alcohol intake across groups but women who separated or divorces and women who remained unmarried from having been separated/divorces showed reductions in alcohol intake compared to women remaining married. In sensitivity analyses that additionally controlled for baseline lifestyle factors, waist circumference, self-rated general health, depression and quantity (for variety outcomes) or variety (for quantity), results were similar to the main models presented here (data not shown). Adjusting for changes in body weight did not appreciably alter estimated changes in diet (Supplementary Table S3). The prevalence of obesity, diabetes, cardiovascular disease and cancer varied somewhat among marital transition groups (Supplementary Table S4), so further analyses were conducted in which participants with these conditions were excluded. Analysis of change in fruit intake by marital transition under these different exclusion criteria (Supplementary Table S5) revealed minimal differences for most estimates, and only men who became widowed showed significant declines in intake across all analyses.

## 4 Discussion

### 4.1 Summary of findings

In a population-based cohort of older adults in UK, we examined changes in fruit and vegetable intake associated with changes in marital status over approximately four years. This study found a clear and consistent reduction in both the quantity and variety of fruit and vegetable intakes in men who became widowed. However, widowhood among women was not associated with any reductions in quantity and variety of fruits or vegetables. Both men and women showed changes in diet associated with separation and divorce, but declines were only in fruit and vegetable variety not in total quantity. Overall, the findings supported our hypothesis that marital terminations would be associated with more negative impacts on men compared to women. The amplitude of reductions in both fruit and vegetable variety and quantity may be clinically meaningful. Previous analyses across 10 EPIC study countries show that lower variety and quantity of fruit and vegetable intake were associated with an increased risk of certain cancers and type 2 diabetes (Cooper et al., 2012, Jeurnink et al., 2012). With respect to quantity, our results showed a combined average decrease of 90 g/d in fruits and vegetables, which is slightly more than one UK portion (80 g) of fruit or vegetable and constitutes about one quarter of the recommended 400 g per day of fruits and vegetables that people should consume. A study by Crowe et al. (2011) in the EPIC-Heart study suggested that each additional 80 g portion of fruit and vegetable consumed per day was associated with a 5 percent reduction in risk of fatal ischemic heart disease. More recent evidence from the Health Survey for England shows that survival increases somewhat monotonically with increasing portions of fruits and vegetables (Oyebode et al., 2014).

### 4.2 Findings in relation to previous work

Previous studies indicate that marital termination has adverse dietary effects in men, particularly decreased fruit and vegetable intakes (Conklin et al., 2014a, Eng et al., 2005, Horwath, 1989, Donkin et al., 1998). In addition, similar to our findings, research has shown that men who are single have less healthy dietary behaviours (Eng et al., 2005, Donkin et al., 1998), with non-married men reporting much lower variety of fruit and vegetable intakes than non-married women (Conklin et al., 2014a). The patterns of associations observed in this prospective study are consistent with the findings reported by Eng and colleagues (Eng et al., 2005) who demonstrated that employed men increase healthy dietary behaviour after remarriage. The results of our study are also similar to the reporting by Lee and colleagues (Lee et al., 2005). of adverse dietary effects in separated/divorced women, and increases in the amount of vegetable consumed in women who enter married over follow-up, compared to women who remained married. The findings of our study are also consistent with earlier work showing that associations between living arrangement and healthy dietary behaviours were stronger for men than women (Roos et al., 1998, Donkin et al., 1998, Gove, 1973, Umberson, 1992).

The finding of gender-specific patterns of both health-promoting and health-damaging associations with marital terminations or remaining unmarried, might be explained by a number of sociological factors underpinning social relationships, such as personal expectations and expected norms for gender roles (Roos et al., 1998, Gove, 1972, Umberson, 1987, Maeland and Aaroe, 1993). Durkheim has proposed that personal relationships provide a healthy environment because marriage confers individuals with a sense of meaning and obligation in life and these social and psychological factors facilitate a person's motivation to engage in healthful behaviours (Umberson, 1987, Maeland and Aaroe, 1993). These factors, moreover, contribute to social support and control from family and wife or husband, which further affect individuals' willingness and responsibility to adhere to shared norms and healthy behaviours (Umberson, 1987, Hahn, 1993).

Accordingly, in the absence of spousal-facilitated social control, the probability of engaging in health-compromising behaviour increases. However, women and men differ in their experience of the social processes underpinning the influence of marriage on health and healthy behaviours. Women are socialised from early adulthood to monitor their own health, and the health and well-being of others which is less common among men (Roos et al., 1998, Gove, 1973). Thus, in a marital relationship, gender determines an asymmetry in spousal influence on health and health behaviours, with men gaining more health benefit from marriage than women (Umberson, 1992).

More specifically, this study found that marital terminations in men, particularly becoming widowed, had a negative impact on healthy dietary behaviours, with the reductions being most pronounced and consistent for variety of fruits and vegetables. In addition, earlier work has indicated that women's dietary behaviours are less altered by widowhood or remaining unmarried (Donkin et al., 1998, Umberson, 1987, Schäfer et al., 1999). These diverse sociological drivers also help to explain how entering marriage showed relative improvements in healthy dietary behaviours in men more consistently than women for whom diets may be more constrained by marriage. The latter is supported by the consistent pattern seen in women of health-protecting dietary effects of widowhood and potentially health-damaging or limited dietary effects of entering marriage (the reverse was seen in men). As argued by Gove et al. (Gove and Shin, 1989), widowed women may have higher levels of life satisfaction, happiness and well-being than men, as men are less prepared for widowhood (Gove and Shin, 1989). Nevertheless, we also acknowledge that spousal influence is bidirectional and women too are influenced by their husbands, as demonstrated by a previous study in Australia (Horwath, 1989).

It is notable, moreover, that the results for divorced versus becoming widowed differed especially for women, showing an opposing pattern of association with each indicator of healthy eating. For men, these two marital transitions did not differ except in relation to fruit quantity. The reasons for the differences in dietary effects between divorce and becoming widowed among women could be manifold. In many cases, divorce or separation is a choice, unlike widowhood. For women, there are likely to be more negative social and, particularly economic, implications of chosen (vs. not chosen) marital termination that constrain women's ability to engage in healthy eating behaviours.

### 4.3 Methodological considerations and limitations

Several limitations are acknowledged. Results are based on self-reported data in which the accuracy of information from questionnaires is variable and prone to recall bias. Also, the degree and type of biases in self-reported dietary questionnaires are likely to vary by gender due to differences in social desirability and social approval (Hebert et al., 1997). However, potential systematic differences were examined suggesting no evidence of non-differential misclassification and a limited risk of bias. We also note that there were low numbers of participants in the transitions of becoming separated or divorced, widowed or getting married, which made the estimates more unstable. Furthermore, our longitudinal analysis does not preclude the possibility of selection bias from individuals with unhealthy habits or risky behaviours who may be selected into remaining single.

The study was further subject to limitation from lack of information on the timing of marital transitions, the length and quality of marriage, and reasons for termination. Thus, dietary effects of marital termination may differ for participants whose relationship ended more than a year ago rather than those who had a more recent experience. This study also did not include other types of social relationships, such as family and friend contact, which might modify the effects of marital transitions on healthy eating as suggested by previous work in this cohort (Conklin et al., 2014a). Finally, although the cohort shared similar characteristics to the general UK population, it was not ethnically diverse (Day et al., 1999). Hence the findings of this analysis are most easily generalised to other older adult white-European populations.

The novelty and strengths of this study deserve attention. We examined a large sample with information on multiple known confounders, including other lifestyle factors, selected from an established population-based cohort study of nutrition and health. We also reported four different aspects of healthy dietary behaviours that are independently associated with reduced disease risk (Cooper et al., 2012, Jeurnink et al., 2012). This study went further than examining only the amount of intake of fruits and vegetables, but also considered variety of intake since variety is an established concept in dietary recommendations (World Health Organization, 2004), is a good marker of overall diet quality (Drewnowski et al., 1997, Bernstein et al., 2002), and varies more by socioeconomic indicators than quantity (Conklin et al., 2014b). Moreover, the gender perspective is a particular strength and novel contribution of the study as it extends the strength and validity of prior research in occupational cohorts (Eng et al., 2005, Lee et al., 2005), and addresses an identified knowledge gap (Braveman et al., 2011).

## 5 Conclusion

This epidemiological study is the first to examine the relationship between marital transitions and change in healthy dietary behaviours using longitudinal data on men and women in the same population-based cohort. Becoming widowed was associated with a significantly decrease in the quantity and variety of fruits and vegetables in men, while men who were separated or divorced or remained unmarried reported a decrease in all dietary indicators except fruit quantity. Weaker associations were seen in women, and only in relation to variety outcomes. Further work is needed on possible mechanisms involving sociological and psychological factors that are likely to explain these gender differences. Future public health interventions to promote health through diets depend on an improved understanding of this critical social determinant.

## Figures and Tables

**Table 1a:** Sociodemographic characteristics and dietary behaviours across marital transitions for men in the EPIC-Norfolk study (n = 4976).

	Marital transitions				
	Remained married	Separated/divorced	Became widowed	Became married	Remained unmarried
N=	4328	52	67	78	451
**Baseline characteristics**					
Age at recruitment, years (sd)	60 (9)	56 (8)	66 (8)	57 (9)	60 (10)
Lowest social class^a^, no (%)	589 (14)	8 (16)	7 (11)	11 (14)	97 (23)
Lowest education^b^, no (%)	1121 (26)	11 (21)	19 (28)	22 (28)	159 (35)
Ever smoker, no (%)	2729 (64)	32 (62)	44 (57)	276 (63)	46 (70)
Body weight, mean kg (sd)	80 (11)	78 (10)	79 (11)	81 (11)	79 (12)
Body mass index, mean kg/m^2^ (sd)	26.3 (3.0)	25.9 (3.5)	26.2 (3.3)	26.4 (2.9)	26.3 (3.7)
In moderate/poor self-reported health, no (%)	642 (14.8)	5 (9.6)	14 (20.9)	11 (14.1)	104 (23.1)
Self-reported depression, no (%)	359 (8.3)	9 (17.3)	2 (3.0)	2 (2.6)	50 (11.1)
Total energy intake, mean kcal/d (sd)	2219 (611)	2180 (498)	2064 (583)	2166 (520)	2206 (701)
Percent energy from protein, mean (sd)	15.9 (2.8)	15.6 (2.2)	16.2 (3.4)	15.9 (2.4)	15.6 (3.0)
Percent energy from carbohydrates, mean (sd)	49.9 (6.6)	49.1 (7.2)	48.9 (7.0)	49.2 (5.9)	49.6 (7.0)
Percent energy from fat, mean (sd)	33.5 (5.7)	34.3 (6.1)	33.5 (5.8)	33.1 (5.8)	33.7 (6.2)
Quantity in fruit intake, mean g/d (sd)	221 (164)	203 (189)	208 (152)	250 (203)	219 (162)
Variety in fruit intake, no/month (sd)	6.7 (2.5)	6.5 (2.5)	6.2 (2.6)	6.8 (2.6)	6.1 (2.5)
Quantity in vegetable intake, mean g/d (sd)	262 (118)	269 (94)	240 (125)	283 (128)	226 (137)
Variety in vegetable intake, no/month (sd)	16.4 (3.9)	16.7 (3.4)	15.0 (3.9)	17.4 (3.7)	13.9 (4.5)
**Changes in weight and diet**					
Change in body weight^c^, mean kg/y (sd)	0.38 (1.09)	0.35 (1.39)	0.10 (1.07)	0.53 (1.06)	0.40 (1.26)
Change in fruit quantity, mean g/d (sd)	14 (151)	10 (165)	−33 (140)	28 (153)	3 (151)
Change in fruit variety, no/month (sd)	0.1 (2.1)	−0.3 (2.6)	−0.4 (2.1)	−0.1 (1.8)	−0.1 (1.9)
Change in vegetable quantity, mean g/d (sd)	−3 (107)	−42 (106)	−21 (111)	5.2 (123)	−1 (122)
Change in vegetable variety, no/month (sd)	−0.1 (2.8)	−1.7 (4.5)	−1.5 (3.7)	0.0 (3.3)	−0.1 (3.0)

All measurements taken at baseline (first health check) except for changes in weight and dietary parameters, which were calculated by subtracting baseline from follow-up values.

a Lowest social classes comprise partly skilled and unskilled occupations.

b Adults with no qualification.

c Change in body weight estimated per year, accounting for the interval between the measurement time points.

**Table 1b:** Sociodemographic characteristics and dietary behaviours across marital transitions for women in the EPIC-Norfolk study (n = 6601).

	Marital transitions				
	Remained married	Separated/divorced	Became widowed	Became married	Remained unmarried
N=	4884	90	204	93	1330
**Baseline Characteristics**					
Age at recruitment, years (sd)	57 (8)	53 (8)	65 (8)	55 (9)	62 (9)
Lowest social class^a^, no (%)	686 (14)	16 (18)	43 (21)	16 (18)	212 (17)
Lowest education^b^, no (%)	1781 (36)	28 (31)	96 (47)	31 (33)	496 (37)
Ever smoker, no (%)	1858 (38)	44 (50)	39 (45)	41 (45)	581 (44)
Body weight, mean kg (sd)	67.2 (11.0)	67.2 (12.2)	66.8 (11.4)	65.7 (9.5)	67.3 (12.1)
Body mass index, mean kg/m^2^ (sd)	25.8 (4.0)	26.0 (4.1)	26.0 (4.1)	25.1 (3.7)	25.9 (4.3)
In moderate/poor self-reported health, no (%)	731 (15.0)	19 (21.1)	40 (19.6)	11 (11.8)	244 (18.3)
Self-reported depression, no (%)	814 (16.7)	21 (23.3)	33 (16.2)	19 (20.7)	261 (19.7)
Total energy intake, mean kcal/d (sd)	1934 (516)	1913 (623)	1982 (549)	1867 (537)	1951 (563)
Percent energy from protein, mean (sd)	17.4 (3.1)	17.3 (3.6)	17.0 (3.0)	17.5 (3.3)	16.9 (3.1)
Percent energy from carbohydrates, mean (sd)	51.5 (6.1)	50.0 (6.7)	50.9 (6.1)	51.4 (6.9)	51.8 (6.6)
Percent energy from fat, mean (sd)	32.2 (5.8)	33.1 (6.5)	33.5 (5.9)	31.9 (6.0)	32.9 (6.3)
Quantity in fruit intake, mean g/d (sd)	279 (184)	251 (173)	280 (162)	282 (191)	303 (211)
Variety in fruit intake, no/month (sd)	7.7 (2.2)	7.5 (2.4)	7.8 (2.3)	7.6 (2.2)	7.6 (2.3)
Quantity in vegetable intake, mean g/d (sd)	290 (135)	264 (111)	289 (131)	279 (114)	281 (153)
Variety in vegetable intake, no/month (sd)	17.1 (3.8)	17.2 (4.2)	16.5 (4.0)	17.6 (3.9)	16.3 (4.3)
**Changes in weight and diet**					
Change in body weight^c^, mean kg/y (sd)	0.40 (1.23)	0.29 (1.33)	0.07 (1.34)	0.51 (1.79)	0.37 (1.16)
Change in fruit quantity, mean g/d (sd)	13 (180)	25 (240)	32 (157)	−14 (178)	5 (217)
Change in fruit variety, no/month (sd)	0.0 (1.9)	−0.3 (2.2)	0.1 (2.0)	0.1 (2.1)	−0.1 (1.9)
Change in vegetable quantity, mean g/d (sd)	−8 (119)	−8 (100)	2 (155)	2 (129)	−10 (137)
Change in vegetable variety, no/month (sd)	−0.1 (2.7)	−0.8 (3.6)	−0.3 (3.5)	−0.3 (2.8)	−0.5 (3.0)

All measurements taken at baseline (first health check) except for changes in weight and dietary parameters, which were calculated by subtracting baseline from follow-up values.

a Lowest social classes comprise partly skilled and unskilled occupations.

b Adults with no qualification.

c Change in body weight estimated per year, accounting for the interval between the measurement time points.

**Table 2:** Mean (95% CI) change in fruit intake in men and women, by marital transition category, referenced to those who remained married. Changes are estimated without covariate adjustment and with progressive adjustment for potential confounders. Data from the EPIC-Norfolk sample (n = 11 577).

Sample	Marital transition	n	Change and 95% CI in fruit intake (g/day) over follow-up period				
Unadjusted	Age adjusted	+ Change in dietary energy	+ Educational attainment	+ Baseline quantity
Men	Separated/divorced	52	−3.0 (−44.3, 38.3)	−5.8 (−47.1, 35.6)	−5.9 (−46.7, 35)	−6.0 (−46.8, 34.8)	−11.3 (−48.5, 25.8)
Became widowed	67	−46.0 (−82.5, −9.6) *P* = 0.013	−41.7 (−78.3, −5.1) *P* = 0.025	−39.7 (−75.9, −3.6) *P* = 0.031	−39.3 (−75.4, −3.2) *P* = 0.037	−47.7 (−80.6, −14.9) *P* = 0.004
Became married	78	14.1 (−19.8, 47.9)	12.2 (−21.6, 46.0)	11.3 (−22.2, 44.7)	11.1 (−22.3, 44.5)	23.9 (−6.6, 54.3)
Remained unmarried	451	−10.6 (−25.3, 4)	−10.4 (−25, 4.2)	−8.1 (−22.6, 6.4)	−8.7 (−23.1, 5.8)	−9.5 (−22.7, 3.7)
Women	Separated/divorced	90	11.4 (−27.9, 50.6)	7.6 (−31.7, 46.9)	8.2 (−29.7, 46.2)	8.4 (−29.6, 46.3)	−1.2 (−35.6, 33.1)
Became widowed	204	18.9 (−7.5, 45.3)	25.5 (−1.2, 52.1)	24.3 (−1.5, 50.0)	24.5 (−1.2, 50.3)	21.6 (−1.7, 44.9)
Became married	93	−27.3 (−65.9, 11.3)	−29.0 (−67.6, 9.6)	−22.9 (−60.2, 14.4)	−22.5 (−59.8, 14.8)	−22.2 (−55.9, 11.6)
Remained unmarried	1330	−8.7 (−20.1, 2.7)	−4.6 (−16.2, 7.1)	−0.7 (−12.0, 10.6)	−0.1 (−11.4, 11.2)	5.9 (−4.3, 16.2)

*P*-Values provided from linear regression comparing each group to those of the same gender remaining married.
