# Supplementary material for: Marital transitions and associated changes in fruit and vegetable intake: Findings from the population-based prospective EPIC-Norfolk cohort, UK
Source: Soc Sci Med. 2016 May;157:120–6. doi: 10.1016/j.socscimed.2016.04.004 (PMC4857700; doi:10.1016/j.socscimed.2016.04.004)
Supplement: Supplementary file 1 [file mmc1.docx]

**Figure S1:** Flow diagram for sample restriction in the European Prospective Investigation of Cancer (EPIC) Norfolk cohort for analyses reported in this study


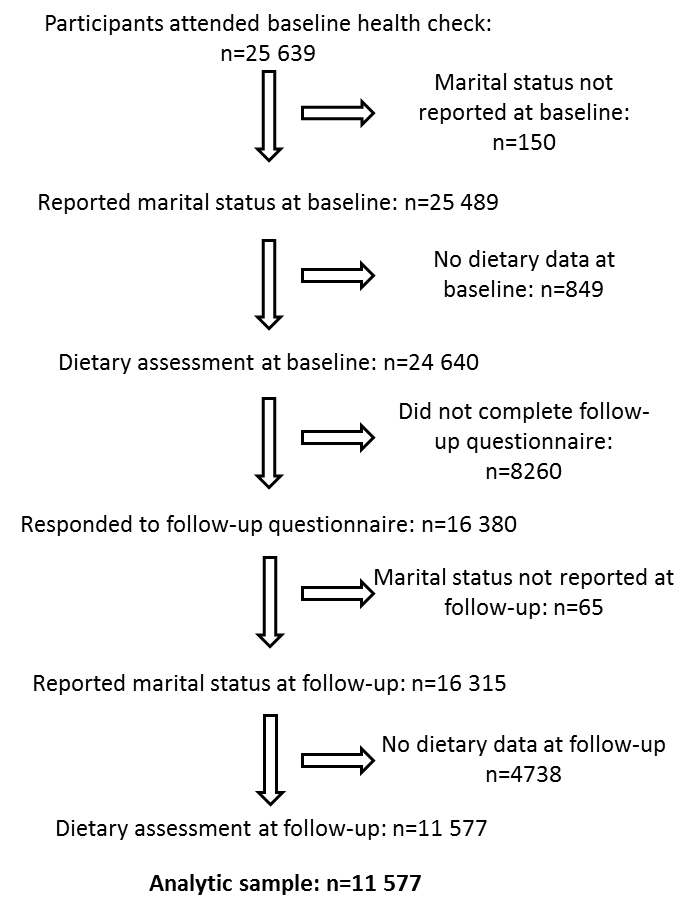


| **Table S1.** Comparison of baseline characteristics of the full EPIC-Norfolk cohort and the analytic sample of adults in the analysis of marital transition, by gender**.** | | | | | | |
| --- | --- | --- | --- | --- | --- | --- |
|  | **full baseline sample**  **n=25 639** | | |  | **analytic sample**  **n=11 577** | |
|  | **Men** | | **Women** |  | **Men** | **Women** |
|  | *n=11 607* | | *n=14 032* |  | *n=4976* | *n=6601* |
|  | (45%) | | (55%) |  | (43%) | (57%) |
| Mean Age (s.d.) | 59.6 (9.3) | | 58.9 (9.3) |  | 60.1 (8.8) | 58.4 (8.8) |
| Married^a^ | 87% | | 76% |  | 89% | 78% |
| Education to degree^b^ | 15% | | 11% |  | 17% | 13%^b^ |
| Higher social classes^c^ | 45% | | 40% |  | 49% | 44% ^c^ |
| Moderate/poor health^d^ | 20% | | 20% |  | 16% | 16% |
| Current smoker^e^ | 12% | | 11% |  | 9% | 8%^d^ |
| Mean BMI (s.d.) ^f^ | 26.5 (3.3) | | 26.2 (4.4) |  | 26.3 (3.1) | 25.8 (4.1) |
| Baseline diet^g^ | |  | |  |  |  |
| Energy intake, kcal/d | 2190 (628) | | 1926 (546) |  | 2215 (617) | 1937 (529) |
| Percent energy from |  | |  |  |  |  |
| Protein | 15.9 (2.9) | | 17.3 (3.2) |  | 15.8 (2.8) | 17.3 (3.1) |
| Carbohydrates | 49.5 (7.0) | | 51.3 (6.5) |  | 49.9 (6.6) | 51.5 (6.2) |
| Fat | 33.7 (5.8) | | 32.6 (6.0) |  | 33.5 (5.7) | 32.4 (5.9) |
| Fruit intake |  | |  |  |  |  |
| Quantity, g/d | 212 (164) | | 278 (201) |  | 221 (165) | 283 (189) |
| Variety, items/m | 6.2 (2.8) | | 7.2 (2.7) |  | 6.6 (2.5) | 7.7 (2.2) |
| Vegetable intake |  | |  |  |  |  |
| Quantity, g/d | 255 (123) | | 284 (143) |  | 259 (120) | 287 (139) |
| Variety, items/m | 15.3 (5.0) | | 16.1 (5.0) |  | 16.2 (4.1) | 16.9 (3.9) |
| Alcohol intake, units/w | 10.3 (11.8) | | 4.5 (5.7) |  | 10.2 (11.4) | 4.6 (5.6) |
| **^a^** For marital status, 150 missing from full sample, none missing from analytic sample; **^b^** For educational qualifications, 18 missing from full sample, none missing from analytic sample; **^c^** Higher social classes defined as those participants in professional or managerial and technical professions. Number missing = 570 from full sample, 174 missing from analytic sample ; **^c^** For health status, 314 missing from full sample, 123 missing from analytic sample; **^d^** For current smoker, 220 missing from full sample, 83 missing from analytic sample; **^e^** for BMI, 54 missing from full sample, 13 missing from analytic sample; For dietary measures, 857 missing from full sample, none missing from analytic sample. | | | | | | |

| **Table S2.** Mean (95% CI) estimated changes^a^ in dietary behaviours in men, by marital transition category, referenced to those who remained married. Changes are estimated adjusted for age, educational attainment, change in energy intake (except the energy intake model) and the baseline value of each variable. Data from the EPIC-Norfolk sample. Bold values indicate statistically significant differences from those remaining married at P < 0.05. | | | | | | | |
| --- | --- | --- | --- | --- | --- | --- | --- |
|  | | Marital Transitions, Men (N=4976) | | | | | |
|  | | Separated/ divorced | Became widowed | Got  Married | Remaining single from reported marital status at baseline | | |
|  |  |  |  |  | Separated/  divorced | Widowed | Single |
| Change in | **n =** | 52 | 67 | 78 | 155 | 114 | 182 |
| Energy intake kcal/d | | -19  (-152, 114) | -108  (-226, 10) | -4  (-113, 104) | -54  (-132, 24) | -**134**  **(-226, -43)** | -15  (-87, 57) |
| Percent energy from protein | | -0.64  (-1.36, 0.08) | -0.51  (-1.15, 0.13) | 0.4  (-0.19, 0.99) | -0.05  (-0.47, 0.38) | 0.42  (-0.08, 0.91) | -0.04  (-0.43, 0.35) |
| Percent energy from carbohydrate | | -1.13  (-3.01, 0.75) | -0.05  (-1.72, 1.61) | 0.33  (-1.21, 1.87) | -0.84  (-1.94, 0.27) | -0.03  (-1.32, 1.27) | -0.59  (-1.61, 0.43) |
| Percent energy from fat | | -0.75  (-2.36, 0.85) | -0.13  (-1.55, 1.29) | -0.47  (-1.79, 0.84) | 0.37  (-0.57, 1.31) | **1.25**  **(0.14, 2.35)** | 0.92  (0.05, 1.79) |
| Fruit quantity, mean g/d | | -11.3  (-48.5, 25.8) | **-47.8**  **(-80.6, -14.9)** | 23.9  (-6.6, 54.3) | -7.6  (-29.4, 14.2) | -9.9  (-35.4, 15.6) | -10.8  (-31.0, 9.4) |
| Fruit variety, no/month | | **-0.64**  **(-1.16, -0.12)** | **-0.62**  **(-1.08, -0.16)** | -0.06  (-0.48, 0.37) | -0.29  (-0.60, 0.01) | -0.16  (-0.52, 0.19) | **-0.45**  **(-0.73, -0.16)** |
| Vegetable quantity, mean g/d | | **-35.0**  **(-60.8, -9.3)** | **-27.7**  **(-50.5, -4.9**) | 17.6  (-3.5, 38.7) | -11.1  (-26.2, 4) | -8.2  (-25.9, 9.6) | -9.4  (-23.4, 4.7) |
| Vegetable variety, no/month | | **-1.61**  **(-2.35, -0.88)** | -1**.57**  **(-2.22, -0.92)** | 0.31  (-0.29, 0.91) | -0.30  (-0.73, 0.13) | **-0.69**  **(-1.2, -0.19)** | **-0.70**  **(-1.10, -0.30)** |
| Alcohol intake, units/week | | 0.64  (-1.13, 2.42) | 0.82  (-0.72, 2.35) | 0.40  (-1.02, 1.82) | 0.31  (-0.71, 1.34) | 0.98  (-0.23, 2.19) | -0.35  (-1.31, 0.60) |
|  | |  |  |  |  |  |  |
| ^a^Linear regression models included the following covariates: age (continuous), change in dietary energy intake (continuous), educational attainment (categorical), baseline quantity in change-in-quantity analyses or baseline variety in change-in-variety analyses (continuous). | | | | | | | |

| **Table S2 (Continued).** Mean (95% CI) estimated changes in dietary behaviours in women, by marital transition category, referenced to those who remained married. Changes are estimated adjusted for age, educational attainment, change in energy intake (except the energy intake model) and the baseline value of each variable. Data from the EPIC-Norfolk sample. Bold values indicate statistically significant differences from those remaining married at P < 0.05. | | | | | | | |
| --- | --- | --- | --- | --- | --- | --- | --- |
|  | | Marital Transitions, Women (N=6601) | | | | | |
|  | | Separated/ divorced | Became widowed | Got  Married | Remaining single from reported marital status at baseline | | |
|  |  |  |  |  | Separated/  divorced | Widowed | Single |
| Change in | **n =** | 90 | 204 | 93 | 426 | 628 | 276 |
| Energy intake kcal/d | | -14 (-99, 72) | 26 (-32, 84) | **-85 (-170, -1)** | **-44 (-85, -3)** | **-38 (-74, -2)** | -4 (-54, 47) |
| Percent energy from protein | | **0.81**  **(0.21, 1.41)** | -0.3  (-0.71, 0.1) | -0.21  (-0.8, 0.37) | -0.1  (-0.39, 0.18) | -0.15  (-0.4, 0.1) | -0.33  (-0.67, 0.02) |
| Percent energy from carbohydrate | | -0.43  (-1.9, 1.04) | **1.24**  **(0.24, 2.24)** | 0.36  (-1.08, 1.8) | 0.58  (-0.12, 1.28) | 0.19  (-0.42, 0.81) | **0.87**  **(0.02, 1.73)** |
| Percent energy from fat | | 0.64  (-0.59, 1.87) | 0.01  (-0.83, 0.85) | 0.4  (-0.81, 1.61) | 0.45  (-0.14, 1.03) | -0.24  (-0.75, 0.28) | 0.21  (-0.51, 0.93) |
| Fruit quantity, mean g/d | | -1.5  (-35.9, 32.9) | 22.1  (-1.2, 45.4) | -22.3  (-56.1, 11.4) | 4.3  (-12.1, 20.6) | 12.9  (-1.5, 27.4) | -6.1  (-26.2, 14) |
| Fruit variety, no/month | | -0.29  (-0.65, 0.08) | 0.15  (-0.1, 0.39) | 0.13  (-0.23, 0.49) | -0.02  (-0.2, 0.15) | -0.09  (-0.25, 0.06) | -0.2  (-0.41, 0.01) |
| Vegetable quantity, mean g/d | | -8.6  (-30.5, 13.4) | 7  (-7.9, 22) | 9  (-12.6, 30.6) | -6.9  (-17.3, 3.6) | -3  (-12.2, 6.2) | -9.5  (-22.3, 3.4) |
| Vegetable variety, no/month | | **-0.74**  **(-1.28, -0.2)** | -0.23  (-0.6, 0.13) | 0  (-0.54, 0.53) | **-0.36**  **(-0.61, -0.1)** | **-0.49**  **(-0.72, -0.26)** | **-0.42**  **(-0.74, -0.1)** |
| Alcohol intake, units/week | | **-1.13**  **(-1.87, -0.38)** | -0.24  (-0.75, 0.26) | -0.09  (-0.81, 0.62) | **-0.5**  **(-0.85, -0.15)** | -0.22  (-0.53, 0.09) | 0.16  (-0.27, 0.59) |
|  | |  |  |  |  |  |  |
| ^a^Linear regression models included the following covariates: age (continuous), change in dietary energy intake (continuous), educational attainment (categorical), baseline quantity in change-in-quantity analyses or baseline variety in change-in-variety analyses (continuous). | | | | | | | |

| **Table S3.** Sensitivity analyse further adjusting for body weight changes occuring over follow-up period, Men (n=4950)^a^  Bold values indicate statistically significant differences from those remaining married at P < 0.05. | | | | | |
| --- | --- | --- | --- | --- | --- |
|  | **Marital Transition** | **n** | **Change and 95% CI in diet over follow-up period** | | |
|  |  |  | **Multivariable model**^b^ | **+ adjustment for change in body weight** |  |
| Change in fruit intake (g/d) | Separated/ divorced | 51 | -13.0 (-50.5, 24.6) | -13.3 (-50.9, 24.2) |  |
|  | Became widowed | 66 | **-44.8 (-77.9, -11.6)** | **-45.5 (-78.6, -12.4)** |  |
|  | Became married | 78 | 23.8 (-6.7, 54.2) | 24.2 (-6.2, 54.6) |  |
|  | Remained unmarried | 441 | -9.4 (-22.6, 3.9) | -9.3 (-22.5, 3.9) |  |
|  |  |  |  |  |  |
| Change in veg intake (g/d) | Separated/ divorced | 51 | **-35.2 (-61.2, -9.1)** | **-35.4 (-61.4, -9.3)** |  |
|  | Became widowed | 66 | **-27.4 (-50.3, -4.4)** | **-27.8 (-50.8, -4.8)** |  |
|  | Became married | 78 | 17.5 (-3.6, 38.6) | 17.7 (-3.4, 38.8) |  |
|  | Remained unmarried | 441 | **-9.5 (-18.7, -0.2)** | **-9.4 (-18.6, -0.2)** |  |
|  |  |  |  |  |  |
| Change in fruit variety (no/month) | Separated/ divorced | 51 | **-0.69 (-1.22, -0.17)** | **-0.69 (-1.22, -0.17)** |  |
|  | Became widowed | 66 | **-0.62 (-1.09, -0.16)** | **-0.63 (-1.09, -0.16)** |  |
|  | Became married | 78 | -0.06 (-0.48, 0.37) | -0.05 (-0.48, 0.37) |  |
|  | Remained unmarried | 441 | **-0.32 (-0.5, -0.13)** | **-0.32 (-0.5, -0.13)** |  |
|  |  |  |  |  |  |
| Change in veg variety (no/month) | Separated/ divorced | 51 | **-1.69 (-2.43, -0.95)** | **-1.68 (-2.42, -0.94)** |  |
|  | Became widowed | 66 | **-1.53 (-2.18, -0.88)** | **-1.53 (-2.18, -0.87)** |  |
|  | Became married | 78 | 0.31 (-0.29, 0.91) | 0.31 (-0.29, 0.91) |  |
|  | Remained unmarried | 441 | **-0.53 (-0.8, -0.27)** | **-0.53 (-0.8, -0.27)** |  |
| ^a^Linear regression models based on analytic sample further restricted for cases with data on measured changes in body weight (26 cases lacked body weight change data). ^b^Covariates included in the multivariate model were age (continuous), change in dietary energy intake ( continuous), educational attainment, baseline quantity (continuous) in change-in-quantity analyses or baseline variety (continuous) in change-in-variety analyses. | | | | | |

| **Table S3 (Continued).** Sensitivity analyse further adjusting for body weight changes occuring over follow-up period, Women (n=6572)^a^ Bold values indicate statistically significant differences from those remaining married at P < 0.05. | | | | | |
| --- | --- | --- | --- | --- | --- |
|  | **Marital Transition** | **n** | **Change and 95% CI in diet over follow-up period** | | |
|  |  |  | **Multivariable model**^b^ | **+ adjustment for change in body weight** |  |
| Change in fruit intake (g/d) | Separated/ divorced | 90 | -1.5 (-35.9, 32.9) | -3.3 (-37.6, 31.0) |  |
|  | Became widowed | 203 | 21.0 (-2.4, 44.4) | 18.7 (-4.6, 42.0) |  |
|  | Became married | 93 | -22.5 (-56.2, 11.3) | -21.5 (-55.2, 12.2) |  |
|  | Remained unmarried | 1325 | 5.4 (-4.8, 15.7) | 6.0 (-4.3, 16.2) |  |
|  |  |  |  |  |  |
| Change in veg intake (g/d) | Separated/ divorced | 90 | 1.4 (-23.8, 26.6) | -8.5 (-30.5, 13.5) |  |
|  | Became widowed | 203 | 9.8 (-7.3, 26.9) | 6.6 (-8.3, 21.6) |  |
|  | Became married | 93 | 13.8 (-11, 38.5) | 9.0 (-12.7, 30.6) |  |
|  | Remained unmarried | 1325 | 0.4 (-7.1, 8.0) | -5.7 (-12.3, 0.9) |  |
|  |  |  |  |  |  |
| Change in fruit variety (no/month) | Separated/ divorced | 90 | -0.29 (-0.65, 0.08) | -0.29 (-0.66, 0.07) |  |
|  | Became widowed | 203 | 0.15 (-0.1, 0.4) | 0.14 (-0.11, 0.39) |  |
|  | Became married | 93 | 0.13 (-0.23, 0.49) | 0.13 (-0.23, 0.49) |  |
|  | Remained unmarried | 1325 | -0.1 (-0.21, 0.01) | -0.1 (-0.21, 0.01) |  |
|  |  |  |  |  |  |
| Change in veg variety (no/month) | Separated/ divorced | 90 | **-0.74 (-1.28, -0.20)** | **-0.75 (-1.29, -0.21)** |  |
|  | Became widowed | 203 | -0.25 (-0.62, 0.12) | -0.26 (-0.63, 0.11) |  |
|  | Became married | 93 | 0.00 (-0.54, 0.53) | 0.00 (-0.53, 0.53) |  |
|  | Remained unmarried | 1325 | **-0.43 (-0.59, -0.26)** | **-0.42 (-0.59, -0.26)** |  |
| ^a^Linear regression models based on analytic sample further restricted for cases with data on measured changes in body weight (29 cases lacked body weight change data).  ^b^Covariates included in the multivariate model were age (continuous), change in dietary energy intake ( continuous), educational attainment, baseline quantity (continuous) in change-in-quantity analyses or baseline variety (continuous) in change-in-variety analyses. | | | | | |

| **Supplementary Table 4.** Prevalence of obesity and self-reported chronic disease across marital transitions for men and women in the EPIC-Norfolk study (n=11 577) | | | | | |
| --- | --- | --- | --- | --- | --- |
|  | Marital Transitions | | | | |
|  | Remained married | Separated/ divorced | Became  widowed | Became  married | Remained  unmarried |
| **Men n =** | **4328** | **52** | **67** | **78** | **451** |
| **Obesity**^a^ **and self-reported chronic disease**^b^ |  |  |  |  |  |
| Obesity, no (%)^c^ | 448 (10) | 7 (14) | 8 (12) | 8 (10) | 63 (14) |
| Diabetes, no (%)^d^ | 118 (3) | 1 (2) | 1 (2) | 1 (1) | 15 (3) |
| Cardiovascular disease, no (%)^e^ | 253 (6) | 3 (6) | 5 (8) | 3 (4) | 31 (7) |
| Cancer, no (%)^f^ | 140 (4) | 2 (4) | 3 (5) | 2 (3) | 28 (6) |
|  |  |  |  |  |  |
|  |  |  |  |  |  |
| **Women n =** | **4884** | **90** | **204** | **93** | **1330** |
| Obesity, no (%)^g^ | 665 (14) | 16 (18) | 35 (17) | 9 (10) | 199 (15) |
| Diabetes, no (%)^h^ | 52 (1) | 1 (1) | 2 (1) | 1 (1) | 21 (2) |
| Cardiovascular disease, no (%)^i^ | 73 (2) | 2 (2) | 6 (3) | 3 (3) | 31 (2) |
| Cancer, no (%)^j^ | 311 (6) | 5 (6) | 15 (7) | 3 (3) | 110 (8) |
| ^a^Obesity defined as BMI 30 kg/m^2^ from height and weight measurements taken at baseline (first health check). ^b^ Chronic disease based on response to checklist question phrased as *’Has a doctor ever told you that you have any of the following conditions...*’. ^c^ Missing five cases.  ^d^ Missing three cases. ^e^ Includes experience of myocardial infarction and stroke; missing one case. ^f^ Missing one case.  ^g^ Missing eight cases.  ^h^ Missing three cases. ^i^ Includes experience of myocardial infarction and stroke; missing one case. ^j^ Missing three cases. | | | | | |

| **Supplementary Table 5.** Association between marital transitions and mean (95% CI) change in fruit consumption in men and women, referenced to those who remained married. Changes are estimated adjusting for age, educational attainment, change in energy intake and baseline intake of fruit. Results for full analytic sample are presented for comparison with analyses conducted after exclusions based on measured obesity or chronic disease reported at baseline | | | | | | | |
| --- | --- | --- | --- | --- | --- | --- | --- |
| **Sample** | **Marital Transition** | **Mean change and 95% CI in fruit intake (g/day) over follow-up period** | | | | | |
|  |  | **full analytic sample**  **n=11 577** | **excluding**  **obese**  **n=10 106** | **excluding**  **w/ diabetes**  **n=11 358** | **excluding**  **w/ CVD**  **n=11 150** | **excluding**  **w/ cancer**  **n=10 944** |  |
| **Men** | Separated/ divorced | -11.3 (-48.5, 25.8) | 6.71 (-32.8, 46.2) | -9.2 (-46.8, 28.3) | -10.2 (-48.2, 27.9) | -11.1 (-49.1, 26.8) |  |
|  | Became widowed | -47.7 (-80.6, -14.9)  *P*=0.004 | -49.8 (-84.4, -15.2) *P*=0.005 | -47.4 (-80.5, -14.2) *P*=0.005 | -43.6 (-77.6, -9.6) *P*=0.012 | -45.2 (-78.9, -11.5) *P*=0.009 |  |
|  | Became married | 23.9 (-6.6, 54.3) | 25.1 (-6.6, 56.8) | 21.4 (-9.2, 52.1) | 19.7 (-11.2, 50.5) | 18.4 (-12.4, 49.3) |  |
|  | Remained unmarried | -9.5 (-22.7, 3.7) | -11.4 (-25.5, 2.6) | -7.02 (-20.45, 6.4) | -9.8 (-23.4, 3.8) | -9.8 (-23.4, 3.8) |  |
|  |  |  |  |  |  |  |  |
| **Women** | Separated/ divorced | -1.2 (-35.6, 33.1) | 7.7 (-29.1, 44.5) | 0.9 (-33.6, 35.3) | -2.1 (-36.6, 32.4) | -23.5 (-58.8, 11.8) |  |
|  | Became widowed | 21.6 (-1.7, 44.9) | 15.7 (-9.2, 40.5) | 22.1 (-1.3, 45.4) | 21.6 (-1.8, 45.1) | 19.2 (-5, 43.4) |  |
|  | Became married | -22.2 (-55.9, 11.6) | -24.9 (-59.4, 9.6) | -24.2 (-58.1, 9.6) | -23.6 (-57.6, 10.5) | -19.3 (-53.6, 15) |  |
|  | Remained unmarried | 5.9 (-4.3, 16.2) | 3.3 (-7.5, 14.1) | 4.2 (-6.1, 14.5) | 6.9 (-3.4, 17.1) | 5.7 (-5, 16.4) |  |

*P*-Values provided from linear regression comparing each group to those of the same gender remaining married
